# Are “Alternative to Discipline” Programs for Nurses With Alcohol and Other Drug Challenges Relevant to Global Contexts? A Scoping Review

**DOI:** 10.1111/inm.70024

**Published:** 2025-03-18

**Authors:** Adam Searby, Rachel Shuster, Leila S. Ledbetter, Marissa D. Abram

**Affiliations:** ^1^ Monash University School of Nursing and Midwifery Melbourne Victoria Australia; ^2^ Highmark Inc. Pittsburgh Pennsylvania USA; ^3^ Duke University Medical Center Library & Archives Durham North Carolina USA; ^4^ Duke University School of Nursing Durham North Carolina USA

**Keywords:** alternative to discipline programs, monitoring programs, nurses, professional impairment, substance‐related disorders

## Abstract

Alternative to discipline programs, defined as programs for nurses ‘impaired’ by issues such as alcohol and/or drug use, provide treatment and support to return to the profession. This paper aims to explore alternative to discipline programs for nurses to determine whether these programs are relevant to other geographical contexts. A scoping review was conducted in accordance with the JBI methodology. The PRIMSA‐ScR checklist was used in this scoping review. The databases searched included MEDLINE (PubMed), Embase (Elsevier), Cumulative Index to Nursing and Allied Health Literature (EBSCOhost), and ProQuest Dissertations and Theses Global (ProQuest) from 1984 to 2024. Title and abstract screening was conducted on 1622 papers, resulting in 90 papers for full‐text screening. After full‐text screening, 19 papers met the inclusion criteria for this scoping review. Issues related to the cost of programs, strict requirements for abstinence, and a lack of ‘bespoke’ options that address participant needs were identified in this review and need to be addressed prior to global implementation of these programs. Impairment of nurses due to alcohol and/or drug use threatens workforce sustainability, and without ‘alternatives to discipline’, can mean experience nurses are lost to the profession, often without treatment. Exploration of alterative to discipline programs is essential to ensure that nurses with alcohol and other drug challenges are retained in the profession and receive the treatment required to remain safe practitioners. Likewise, the perception that programs were punitive in nature should be addressed to ensure acceptability of these programs as an alternative to the loss of employment.

## 1 Introduction

Impaired nursing practice can be caused by several reasons, including both physical health and behavioural health challenges, though some of the most common reasons can be attributed to the disordered use of alcohol and/or other drugs. Nurses with substance use disorders (SUDs) may face public disciplinary action, though alternative‐to‐discipline (ATD) programmes can offer a less punitive approach to addressing the safety‐sensitive practice concerns of a nurse with substance use challenges (Bettinardi‐Angres et al., 2012). ATD programmes are monitoring programmes for impairment through alcohol and/or other drug use for licensed, registered, or certified nursing professionals that are both non‐disciplinary and non‐public to address the physical, mental, or behavioural challenges that can or could negatively impact the safety or quality of care being provided by the professional (Bettinardi‐Angres et al., 2012). While both disciplinary and ATD programmes aim to protect public safety through ongoing monitoring, treatment mandates, and other requirements, ATD programmes are often confidential and maintain the objective of safely returning the nurse to practice (Ross et al. 2020). These programmes have been established in many regions of the United States (US) since the 1980s; however, other parts of the world do not have alternative‐to‐discipline programmes, instead relying on registration and regulatory bodies to sanction nurses (Bettinardi‐Angres et al., 2012).

The prevalence of alcohol and other drug use among nurses is poorly defined globally; however, a recent survey found that approximately 18% of nurses in the United States had risky substance use, and 6.6% had a SUD (Trinkoff et al. 2022). Additionally, there are few research studies that explore levels of alcohol and other drug use that cause role impairment. However, statistics do exist, particularly regarding drug diversion (where medications such as opioids are redirected for personal use rather than administered to patients). For example, Gabele et al. (2023) surveyed 172 nurse leaders in California, finding that 65% had experienced a drug diversion investigation, and 64% had experience in supervising a nurse with a known substance use disorder (SUD). Cook's (2013) survey of 119 nurses in a community hospital in the United States found that 74% of respondents had also worked with a nurse with SUD at some point in their career. Although these studies are limited by small sample sizes, potential response bias, and subjective ideas around substance use disorder rather than diagnostic criteria, these findings potentially indicate that substance use disorder is common among nurses.

## 2 Background

According to the National Council of State Boards of Nursing (NCSBN n.d.), ATD programs, when implemented specifically for SUD, “… enhance a [Board of Nursing's] ability to quickly assure public protection by promoting earlier identification, requiring immediate removal from the workplace, and evidence‐based treatment for nurses with substance use disorder,” (para. 1). The first ATD program started in Florida, though several state nursing boards followed in response to the American Nurse's Association's 1982 call for an alternative to address complaints related to alcohol and/or other drug use by nurses using a nonpunitive approach that encouraged treatment before disciplinary action (Mallia 2015). Previously, nurses would face a disciplinary process that was on the public record, being published in the National Practitioner Data Bank, permanently marking their disciplinary record on their nursing licence (‘registration’), including detailed and publicly available documents accounting the action taken against the nurse (Russell, 2020; Shuster 2021).

This response to alcohol and/or other drug use disorders among nurses was likely shaped by drug policy around the globe, such as the “War on Drugs” in the United States, which tended to a punitive, stigmatised, punishment‐based societal opinions of substance use; for example, drug‐related offences resulting in termination of employment, or the inability to obtain suitable employment (Cohen et al. 2024). Further, the opioid crisis occurring in the United States has also resulted in punitive, stigmatising measures to address “misusers” of opioid medications, such as tamper‐proof formulations to prevent crushing and injecting pills, tighter regulations around diversion of medication prescriptions and ‘doctor shopping,’ and other measures that tend to apportion blame to those dependent rather than providing a harm‐reduction focussed approach (Dasgupta et al. 2018; Humphreys et al. 2022).

The shift to a more compassionate, non‐punitive approach was largely due to how the modern disease theory of SUDs was becoming increasingly accepted in the US, with the American Medical Association categorising alcohol use disorder as an illness in 1956 and other addictions as a disease in 1987 (Leshner 1997). In 1996, a workgroup of the NCSBN created a blueprint of the ATD model for other jurisdictions to shape their programmes (Bettinardi‐Angres et al., 2012). Today, 47 of the 59 nursing regulatory bodies in the US have an ATD programme for nurses (NCSBN, n.d.).

Because these programs are built around the stipulations of each jurisdiction's nurse practice act, the differences between ATD programs can vary, from eligibility and exclusion criteria to length of monitoring and specific requirements of participants (Shuster 2021). Some of the nursing regulatory bodies within the US choose to oversee their jurisdiction's ATD, whereas others elect to contract with an external agency (Shuster 2021). Despite differences between jurisdictions, the cornerstone of these ATD programs is that they offer the nurse, or sometimes even nursing students, a confidential, non‐public pathway to maintaining (or working towards eligibility for) their nursing licence and returning to safe nursing practice (NCSBN, n.d.; Shuster 2021).

In terms of entry to alternative discipline programs, literature on substances used or entry leading to program entry is sparse. In a retrospective study of 1533 nurses with impaired practice in Texas, Mumba et al. (2019) found most were in the 30–39 age group (*n* = 508, 33.7%), mostly using opioids (*n* = 451, 29%), followed by alcohol (*n* = 296, 25.5%), with 36% of participants indicating the use of more than one substance. The top four reasons for referral were diversion (*n* = 450, 25.6%), impairment at work (*n* = 311, 20%), arrest (*n* = 307, 19.7%), and a diagnosis of substance use disorder (*n* = 206, 13.2%). Additionally, in a mixed‐methods analysis of nurses' death investigation notes in the United States (*n* = 203), Davidson et al. (2021) found the top four reasons for suicide by nurses were depression (82.3%), use of prescription medications (66%), substance use disorder or misuse (65%), and job loss (61%). Furthermore, 92% (*n* = 187) of these nurses were either out of work or in the process of losing employment. These figures show the importance of ATD programs not only to preserve workforce sustainability but to prevent the loss of life of nurses by suicide.

## 3 Aims

This scoping review aims to explore alternative to discipline or monitoring programs for nurses whose practice is ‘impaired’ due to alcohol and/or other drug use, and given these programs are prevalent in the United States of America, to determine whether these programs are relevant to other geographical contexts. Our secondary aims include to determine common components of these programs, whether these programs are effective in treating problematic alcohol and/or drug use in nurses, and whether they are effective in returning nurses to the workforce (providing an ‘alternative to discipline’).

## 4 Methods

### 4.1 Design

This work is a scoping review. It was carried out using the JBI Manual for Evidence Synthesis (Peters et al. 2015) and was reported following the PRISMA extension for scoping reviews (PRISMA‐ScR) (Tricco et al. 2018). The review was registered under the Open Science Framework (OSF, registration number DOI 10.17605/OSF.IO/WN39Z).

### 4.2 Information Sources

The databases searched included MEDLINE (PubMed), Embase (Elsevier), Cumulative Index to Nursing and Allied Health Literature (EBSCOhost), and ProQuest Dissertations and Theses Global (ProQuest).

### 4.3 Search Strategy

The search was developed and conducted by a professional medical librarian in consultation with the author team and included a mix of keywords and subject headings representing nurses and alternatives to the discipline, respectively. Search hedges or database filters were used to remove publication types such as editorials, letters, case reports, comments, and animal‐only studies as was appropriate for each database. The search was conducted on April 9, 2024, and found 1622 citations. Complete reproducible search strategies, including date ranges and search filters, for all databases are detailed in the Supporting Information. The search strategies for this review are shown in Appendix 1.

### 4.4 Eligibility Criteria

Given the confidential nature of these programs, and the scant literature evaluating or describing them, literature was included if it had been published in the English language and published in an academic journal from 1984 onwards to align with the ANA's resolution to adopt non‐punitive state assistance programs for nurses with impairment from alcohol and/or other drugs (Bettinardi‐Angres et al. 2012). JBI protocol for inclusion criteria, patients, concepts, and context (PCC) provided guidance for reviewers to select sources and for readers to understand the review (Aromataris and Munn 2020).

#### 4.4.1 Participants: Nurses

The participants in this scoping review are nurses: enrolled, registered, licensed, or nurse practitioners.

#### 4.4.2 Concept: Nursing Impairment

Although impaired practice in the nursing context can be caused by mental or physical health conditions, in this review, we consider impairment because of alcohol and/or other drug use disorders; this is not limited to intoxication in the workplace and is a situation where the problematic use of alcohol and/or other drugs has a substantive impact on work performance.

#### 4.4.3 Context: Alternative to Discipline Programs

ATD programs, or non‐disciplinary monitoring programs, are programs that aim to treat and return nurses ‘impaired’ by alcohol and/or other drug use to the nursing workforce in lieu of disciplinary action, such as permanent revocation of their licence/registration. This review focusses specifically on these programs to determine their feasibility for geographic settings outside the United States.

The inclusion criteria for this scoping review were:

1. Papers or studies where registered nurses, nurse practitioners, or licensed practical nurses are the focused population.
2. Papers or studies where nursing programs that cater to nurses who have self‐disclosed or been impaired at work (“alternative to discipline programs,” “monitoring programs”) are the intervention of interest.
3. Papers or studies where the outcome is entrance to programmes for nurses who use alcohol and/or drugs designed to return them to the workforce (i.e., alternative to discipline)
4. Papers using primary research evaluating or providing a thorough description of the ATD or non‐disciplinary monitoring program

The exclusion criteria for this scoping review were:

5 Have a population of interest that is not nurses, that is, other healthcare workers.
6 Have interventions that involve termination of employment or disciplinary measures for reasons other than alcohol or substance use.
7 Have an outcome that is the termination of employment.
8 Editorials, letters, opinion pieces, or commentary.

### 4.5 Selection of Evidence Sources

After the search, all identified studies were uploaded into Covidence (Veritas Health Innovation, Melbourne, Australia), a software system for managing systematic reviews, and duplicates were removed by the software (*n* = 549), with six removed manually. A final set of 1067 citations was left to be screened in the title/abstract phase. Study selection was carried out independently by two authors (author one and author four). Studies were excluded if they did not clearly meet inclusion criteria based on title and/or abstract review.

**FIGURE 1:**
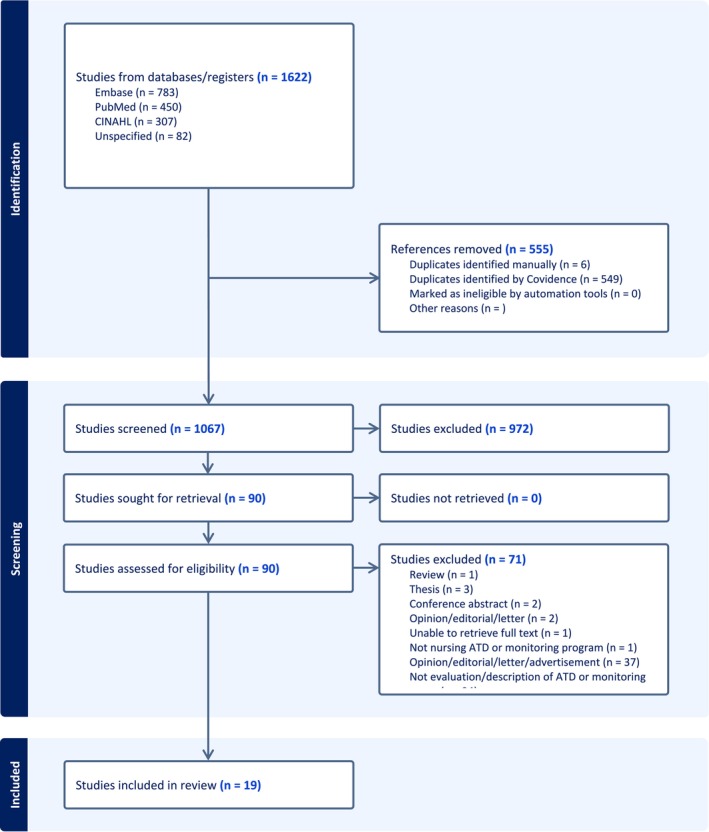
PRISMA study diagram.

For the full‐text screening stage, papers were also reviewed in detail by two independent reviewers and were excluded if they did not meet the inclusion criteria. Any conflicts between the two independent reviewers were resolved through discussion at each stage of the title abstract screening and full‐text selection process. The article selection is presented by flowchart as per PRISMA guidelines (Figure 1).

#### 4.5.1 Data Extraction Process

Data were extracted from included articles by two independent reviewers, using PCC inclusion criteria and the data extraction tool shown in Table 1. The data extraction form was designed in Microsoft Word and pilot tested by two team members and revised for clarity and consistency. Data were extracted from included articles by two independent reviewers, using this finalised form. Any conflicts between the two independent reviewers during the data extraction process were resolved through discussion.

**TABLE 1:** Characteristics of included primary studies.

Author, year	Study aim	Study design and setting	Sample size	Significant findings and outcomes
Choflet et al. 2023	To replicate and evaluate the experience of a nurse with substance use disorder completing a simple internet search for a state alterative to discipline program prior to self‐reporting	Google search of 50 continental US states and Washington DC from April–September 2022	43 states with alternative to discipline programs	Only three websites outlined out of pocket expenses to participants Many links had a perception of ATD programs as punitive; nurses were cautioned against self‐report in these links, and a legal site recommended legal advice before self‐reporting to an ATD program “Monitoring” (service) fees ranged from $0–$175/month, initial evaluation up to $1500, screening (usually 24 tests/year) $35–$100, counselling $100–$200 per session
Fletcher and Ronis 2005	To measure the satisfaction of impaired health professionals with treatment and monitoring programs in Michigan and Indiana	Survey of program participants in Michigan and Indiana	*N* = 383 (168 nurses in Michigan, 120 nurses in Indiana)	Participants generally satisfied with programs Approximately 20% self‐referred to the service Nearly half of program participants lacked insurance coverage for program costs, particularly for toxicology screening Considerable financial distress associated with program and legal costs, in addition to reduced working hours due to sanctions
Fogger and McGuinness 2009	To explore the experiences of nurses in monitoring programs in Alabama	Qualitative survey distributed to monitoring program participants	*N* = 173	Restriction to the ability to work with narcotics noted as a significant barrier to return to work Restriction on overtime shifts was noted as a financial barrier to being able to pay for monitoring program Difficulties in remaining anonymous while in the program 92% of nurses viewed the program as contributing to abstinence from substances
Griffith et al. 2021	To examine the characteristics and outcomes of 189 nursing alternative to discipline program participants in North Carolina	Cross‐sectional retrospective analysis of North Carolina Board of Nursing records	*N* = 189 records	Significant association between program utilisation and mental health diagnosis 72% of participants had familial histories of substance use disorder Program participants primarily worked in hospital settings Primary substances used were opioids such as oxycodone, hydromorphone and hydrocodone
Horton‐Deutsch et al. 2011	To explore nurses' experience in an alternative to discipline treatment program in Indiana	Qualitative focus group study with nurses who had experienced the alternative to discipline program	*N* = 25	“Being accountable” described as the most important aspect of the alternative to discipline program Poor communication on responsibilities and expectations from program staff noted to be a substantial barrier to engagement Participants described a need for monitoring programs to be holistic to address the underlying factors for substance use
Monroe et al. 2013	To estimate the 1‐year prevalence of employed nurses requiring an intervention for substance use in the United States, and the 1‐year prevalence of participation in monitoring programs to compare with the general population	Cross‐sectional retrospective analysis of National Council of State Boards of Nursing data	*N* = 51 US states	Substance use disorders present in 5.1 per 1000 nurses Nurses reported to generally have a rate of substance use disorder similar to the general population
Mozingo 2021	To evaluate the efficacy of an alternative to discipline program in Washington State	Eight‐week monitoring of program completers	*N* = 32 program alumni	81% of nurses returned to work after program completion 90% of program alumni indicated no incidence of relapse Program feedback included a feel that it was punitive, with a lack of recovery focus and a strong emphasis on test results rather than individual progression
Mumba 2018	To describe the lived experiences of nurses with SUD participating in peer assistance programs and the subsequent implications for employment.	Qualitative, phenomenological interviews.	*N* = 10.	Stress from restrictions placed on practice noted, particularly restrictions around handling narcotic drugs. Participants noted significant stigma and shame with being a nurse with substance use disorder. Six participants had a history of substance use prior to entering a peer assistance program.
Ross et al., 2020	To explore nurses' experiences in, and effectiveness, of a Canadian alterative to discipline program	Qualitative, ethnographic interviews; analysis of publicly available documents exploring experiences of alternative to discipline programs	*N* = 12	Participants using the program described it as “unfavourable” for return to practice Program described as punitive with few personalised options for treatment Mandated abstinence meant some options for pharmacological treatment were excluded Outsourcing of program components was believed to create conflicts of interest
Russell 2020	To explore the components and requirements of state nurse monitoring programs to determine the similarities and differences across programs	Document analysis of all alternative to discipline programs in the United States	*N* = 27 programs (69% of all United States Board of Nursing alternative to discipline programs)	Abstinence was a requirement in 85% of programs, as were workplace restrictions (85%) Some program documents indicate that components are individualised to each participant on a case‐by‐case basis
Smiley and Reneau 2020	To assess the SUD program completion rates and determine the program characteristics associated with program completion	Retrospective cohort study of nurses participating in SUD programs between the years 2007 and 2015	*N* = 7737	Bimonthly random drug tests, daily check ins and minimum 3‐year length of stay in alternative to discipline program were associated with successful completion The factor most strongly associated with successful program completion is the number of times the nurse was selected for a drug test A relapse at any time is associated with program non‐completion

## 5 Results

Eleven primary research papers met the inclusion criteria, and six papers that described alternatives to discipline programs were included to provide an overview of program structure, eligibility, and components. Of the primary research papers included in this review, four used quantitative methods (two surveys, a cross‐sectional data analysis and a retrospective data analysis), and the remaining five used qualitative methods. Papers included had diverse aims, including measuring satisfaction with programs, exploring experiences with programs, examining participant characteristics, and assessing program completion rates. Eight studies were conducted in the United States, with one set in Canada. Six papers that described alternatives to discipline programs were also included. Although these papers varied widely in methodology and structure, these papers were descriptive without a research component; however, given the nature of publicly available information on alternatives to discipline programs (i.e., limited), we included them to verify claims made in primary studies, such as program components and cost.

We asked five key questions of the literature on alternatives to discipline programs, specifically related to whether these programs are feasible outside the United States: (1) Does abstinence fit? (2) Is random testing feasible? (3) Is asking nurses to pay for their program realistic? (4) Is there room for a bespoke program? (5) Can accountability be maintained without an alternative to discipline program?

### 5.1 Does Abstinence Fit?

Russell's (2020) overview of state nurse monitoring programs found that abstinence was required in 85% of included programs, and although it was not explicitly stated in the remaining programs, it was implied in the description. This indicates the importance placed on abstinence as a treatment paradigm. The included papers discussing alternatives to discipline programs supported this, with five of the six explicitly describing abstinence as being required. The sixth paper did not specify abstinence; however, it indicated that a ‘zero tolerance’ urine drug screen test regime was required for program completion. Additionally, two programs indicated that program participation included abstinence from alcohol.

Many included papers used the definition of addiction as a ‘disease,’ hence the requirement for abstinence to continue in programs. There were instances where this was noted as detrimental; for instance, a participant in Ross et al.'s (2020) qualitative study of alternative to discipline program participants in Canada found that although pharmacotherapy for alcohol and drug addiction was available, it wasn't promoted to participants as a treatment option. Another participant noted that the ‘premium’ placed on abstinence and 12‐step models led to an expectation that participants would cease the use of medications legitimately prescribed for mental ill health:

[My medical examiner] told me that if I work the steps hard enough I could probably come off my bipolar medication… When my psychiatrist tried to advocate, she was ignored, despite knowing me for years and being a specialist in [the areas of women and health care professionals] with mood disorders… She was told what she could or could not prescribe … (pg. 6)

The focus on addiction as a disease, with a strict abstinence mandate, may preclude addressing the underlying causes of alcohol or substance use disorders. Further, many modern treatment ideologies consider relapse as part of the cycle of change and an opportunity for learning; Smiley and Reneau's (2020) retrospective cohort study of nurses participating in programs (*n* = 7737) found programs had no tolerance for relapse, despite a finding that nurses could complete programs after a relapse, and recommendations that programs provide tailored, gender‐sensitive interventions to improve success rates when relapse occurs.

Although programs requiring abstinence from all substances, including pharmacotherapy, were viewed as punitive in many websites located in the search conducted by Choflet et al. (2023), participants in Fogger and McGuinness' (2009) survey study of 173 nurses involved in monitoring programs noted that 92% of respondents reported that these programs contributed to ongoing abstinence. This is echoed in Horton‐Deutsch et al.'s (2011) qualitative study of 25 nurses participating in an alternative to discipline program in Indiana, where focus group participants described accountability as a key tenet of the program and abstinence as an outcome of this.

ATD programs in the United States ascribe to an abstinence model, as indicated in the programs examined in Table 2. This treatment ideology also extended to the program in Canada described by Ross et al. (2020), where total abstinence from all substances, including those not problematic for the individual, was required. This ideology extended to requiring participants to have a total commitment to a 12‐step model, despite their personal or spiritual beliefs; participants in the study noted that an option for an alternative, ‘non‐secular’ model was touted; however, choosing this option resulted in a breach of contract with the program.

**TABLE 2:** Characteristics of alternative to discipline programs.

Program setting	Program characteristics
Tennessee (Harkreader 2014)	Monitoring 400 healthcare professionals Alternative to discipline model Funded by Tennessee Board of Nursing Abstinence based Active monitoring (drug testing) for “compliance” Privatised components (toxicology screening, online support groups)
Texas (“The Texas Peer Assistance Program for Nurses (TPAPN): A Voluntary Program Playing a Key Role in Maintaining and Regaining Safe Nursing Practice,” 2018)	Voluntary participation: alternative to discipline is based on defined criteria Random monitoring (drug testing) required Participant responsible for all expenses incurred during program (including evaluations, testing, treatment programs, recovery support, aid groups) Abstinence‐based (including alcohol) Prescribed “mood altering… and/or potentially addictive substances” required for treatment may result in exclusion from the program Typical 2‐year contract
Iowa (Beebout and Ruby 2019)	Alternative to discipline program for nurses who “voluntarily submit” Admission requires full disclosure; “denial… or refusing to acknowledge” may result in ineligibility Abstinence‐based (including alcohol) Random testing required Worksite restrictions and monitoring required Typical 3‐year contract
Oregon (Hamilton 2019)	561 nurses have used the program, 2010–2019; 61.5% program completion rate No monitoring cost to the nurse; however, nurses pay for evaluations, treatment and toxicology Four‐year monitoring period Weekly check‐in required Random testing required; minimum 36 tests in the first year
New Jersey (Smith 2013)	Voluntary alternative to discipline program Program includes random urine drug screens, monthly self‐reports, compulsory attendance at 12‐step meetings, return to work and employment oversight Five‐year monitoring period and 6‐months practice required for program completion Abstinence‐based No return to practice until “appropriate treatment” is completed. Approximately 40% of enrolees are working in nursing
Florida (Smith and Greene 2015)	Monitoring agreements range from two to 5 years Random toxicology screening required Weekly attendance at support group and mutual support group meetings required Workplace monitoring required

#### 5.1.1 Is Random Testing Feasible?

Random drug testing, through urine, hair, or blood, was a feature of all six programs evaluated for this review. Russell's (2020) evaluation of the components of 27 alternatives to discipline programs notes that toxicology testing is a component of all programs, used to enforce abstinence, with testing frequency tending to decrease after the first year. It should be noted, however, that this frequency of testing can be almost weekly; for example, a program included in this review conducted in Oregon specifies a minimum of 36 tests in the first year (Hamilton 2019).

Strongly tied to the notion of abstinence at all costs, participants in Mozingo's (2021) study of program alumni found that the program was perceived as punitive, with a strong emphasis on toxicology test results. However, in a retrospective cohort study of 7737 nurses participating in these programs from 2007 to 2015, the factor most strongly associated with program completion was the number of times the nurse was selected for a random drug test (Smiley and Reneau 2020).

None of the papers included in this study specify the substances tested for in random screening; however, the vast majority included in Russell's (2020) component evaluation required the program participant to pay for toxicology testing. As noted in a survey of program participants in Michigan and Indiana, the expectation of payment for program components such as testing creates a burden on participants. Of the 383 included in this study, nearly half lacked insurance coverage for toxicology screening (Fletcher and Ronis 2005).

#### 5.1.2 Is Asking for Nurses to Pay for Their Programme Realistic?

As mentioned in the previous section, program components are at the cost of the nurse contracted to the program; of the six program descriptions we included in this review, all described some form of payment by the participant. In their search of internet resources on alternatives to discipline programs, Choflet et al. (2023) found that only three of 43 program websites explicitly displayed the costs of the program. They noted that these fees ranged from an initial evaluation fee of up to $1500, monitoring or service fees of up to $175/month, testing fees of $35–$100 per test, and counselling session fees of $100–$200 per session.

Participants in Fletcher and Ronis' (2005) qualitative survey of alternative to discipline program participants in Michigan and Indiana reported considerable financial distress with program participation, not only due to the aforementioned costs associated with the program but also legal costs incurred. These costs were compounded by the fact that many participants were either unable to work on enrolment in the program or had significantly reduced working hours and limits on taking on overtime and further work to meet program costs. These concerns are also noted in the qualitative survey of program participants in Alabama (*n* = 173), where it was noted that no overtime was permitted while a nurse was enrolled in a monitoring program, with the authors suggesting that this restriction was lifted to assist in meeting program costs. As a participant in the qualitative study conducted by Horton‐Deutsch et al. (2011) stated:

But a lot of times the nurses are on probation or they temporarily lose their license or whatever, so they really don't even have an income coming in and they're hitting you with all these expenses. You know, you might be talking three, four, five hundred dollars extra every month. (p.452)

According to participants in the qualitative study conducted by Ross et al. (2020), there is a perception that the transfer of components of alternative to discipline programs to private providers resulted in denial of the use of universal healthcare in Canada, and several of the program descriptions examined for this review used private toxicology laboratories at significant cost to participants. Participants in this study described a significant perception of conflict of interest, where medical examiners were charging up to $3000 for assessments while maintaining a financial interest in components of monitoring or alternative to discipline programs:

He got [$3,000 Canadian dollars] … to do his assessment of me. When I returned from treatment, he assessed me again, [$1,000 Canadian dollars] for one hour, and set up my … [return to work] contract, which included mandatory enrolment in [Monitoring Co.], of which he is the medical director and co‐owner That enrolment was $650 [Canadian dollars per] month for 3 years. The first year was paid by [the union], the second 2 years were out of [my] pocket… They make a lot of money off nurses who are in their monitoring programs… It's a massive conflict of interest, and somehow no one addresses it. (p. 8)

#### 5.1.3 Is There Room for a Bespoke Program?

Criticisms of alternative to discipline programs include the notion that they are too standardised, and do not consider personal circumstances. Griffith et al's (2021) analysis of participants (*n* = 189) in an alternative to discipline program in North Carolina found that there was a significant association between program use and mental health diagnosis, along with strong family histories of substance use disorder. Personal histories of substance use were also noted before involvement in the program (Mumba 2018). These findings potentially indicate that ‘one size fits all’ programs, with a focus on monitoring and public safety, may not necessarily be addressing the needs of nurses with problematic alcohol and/or other drug use. Participants in Horton‐Deutsch et al.'s (2011) study, who had experienced alternative to discipline programs supported this notion. As the following participant reported, alternative to discipline programs can be a “blanket” solution to diverse problems:

It seems like it's a blanket solution for a really very complex problem and if you talk to people in ISNAP, everybody's got a different story. (p. 451)

In the reviewed papers there was also a strong focus on the use of 12‐step programs as adjuncts to abstinence and recovery from substance use disorders. These programs typically dictate abstinence, and an acceptance that one has a disease and is powerless over addiction. Although these programs have been successful for many people worldwide, the dominant abstinence/morality discourse embedded in 12‐step models in not appropriate for many participants (Frank 2011). This is reflected in Ross et al.'s (2020) paper, where participants described objection to the 12‐step model, and particularly, the perception that a regulatory body could police their thoughts and spiritual beliefs. These models also guided the treatment paradigms of programs, including perceived ‘character defects’ that led to substance use disorders, as indicated by the following participant in the study:

All of a sudden, people are bombarding you and getting in your face and saying, ‘Have you accepted Step 1 of the 12 steps? Do you realize that you're powerless?’ … We had to do an exercise where we had to identify which character defects, including the seven deadly sins, contributed to our substance use. (p. 5)

A distinct advantage mentioned by participants in the included qualitative studies was the notion of accountability; nurses felt that participation in an alternative to discipline program kept them accountable, which often meant abstinent from alcohol and/or other drugs. For instance, all three focus groups in Horton‐Deutsch et al. (2011) study described accountability as the most import aspect of alternative to discipline programs. For example, a quote from a focus group participant describes this notion, describing accountability to not only remain abstinent from alcohol, but to maintain accountability to the profession:

If it weren't for [program] I would be drinking today. I guarantee it. I think the oversight and knowing that I was on a tight rein, that if I didn't do what I needed to do and at first I didn't think I needed to do it. For me, I just thought I needed to do it to keep my license and my, over time, I've grown into an acceptance of who I am and what I am and what I need to do for me and my health. But I guarantee that if I, especially in those first 2 years, if I had not been under a tight rein I would be drinking today. (p. 450)

The notion of accountability was evident in Mozingo's (2021) study of program completers in Washington State, where 81% of nurses returned to work after program completion, and 90% reporting no relapse. These figures were gained by self‐report and were 8 weeks from program completion, so need to be interpreted with these caveats in mind. However, several web links located by Choflet et al. (2023) indicated the punitive nature of alternative to discipline programs, especially where self‐report was involved. It was felt that self—report also meant mandatory disclosure to nursing regulatory boards and bodies, which often resulted in suspension of employment and legal issues. This was further complicated by the difficulties in remaining anonymous while in programs, including the program in Alabama described by Fogger and McGuinness (2009). In this case, it was noted that anonymity in rural areas is almost impossible; toxicology tests are often conducted within the nurses' employing organisation, and restrictions placed on administering narcotics signalled participation in an alternative to discipline program. Restrictions on narcotic prescribing were reported elsewhere (Mumba et al. 2019; Russell 2020), and although they intend to protect the public and the nurse enrolled in the program, they are described as being a barrier to work rather than attributing to accountability.

## 6 Discussion

This scoping review has explored the concept of ATD or non‐disciplinary monitoring programs for nurses with SUDs, considering whether these programs are relevant beyond North America. Our review of the literature found that although programs were relatively successful in returning nurses to the workforce, there were several considerations to establishing these programs in other jurisdictions. These considerations include a strict mandate on abstinence‐based treatment, the notion that these programs are a ‘no choice,’ punitive method of addressing alcohol and other drug use, personal costs of the program, and that programs operate a strict, standardised model, where non‐adherence to the treatment regime leads a nurse to be labelled as a failure of the program, liable to criminal prosecution, stripping of their registration, and a loss of their livelihood.

A retrospective cohort study of nurses conducted by Smiley and Reneau (2020) who had participated in alternative to discipline programs in the United States in the period 2007 to 2015 found that duration in the program was correlated with successful completion; those who completed the program were found to have a higher number of negative drug screens, greater engagement with program staff, and higher attendance at peer support group meetings. Having a relapse at any time during program completion was associated with non‐completion, indicating that abstinence may be the only accepted criterion for success. Successful completion was determined by each program and not specified in the paper, making it difficult to compare programs. However, it was found that participation in a program for a period of 5 years, with twice‐monthly drug screens, was strongly associated with program completion. The authors suggest a minimum stay of 3 years in the program, with daily check‐ins (including weekends), weekly meetings, and no tolerance for relapse, which reflects participants' comments in some of the qualitative studies included in this review: programs are too restrictive, demand too much of the nurse, and feel punitive in nature.

The notion of abstinence as a measure of success was felt to be a ‘cookie cutter’ approach by Ross et al. (2020) where participants felt they needed to fit in to the program or be deemed to have failed. This was also noted in measures of success; for example, in a meta‐analysis of success rates for substance use disorder monitoring programs, Geuijen et al. (2021) defines success as abstinent and returning to work after treatment. The default position is to assume alcohol and/or other drug use is impairing practice and endangering the public, even if there is no evidence to make this determination. Hence, the only ‘good’ outcome for many programs is abstinence from alcohol and/or drugs.

Of note, in the papers selected for this review, none operated under a harm reduction paradigm, rather operating using a strict abstinence framework as described in the preceding paragraph. Harm reduction, defined as an approach that recognises individuals will continue to use alcohol and/or other drugs and implements interventions which make this use as safe as possible for the individual and society, is also a means to provide care until individuals are ready to make changes to their use of substances (Abram et al. 2025). Not using harm reduction in contemporary alternative to discipline programs arguably makes these punitive; as illustrated in our review, the onus on nurses enrolled in programs to financially support testing to ensure total abstinence places a substantial burden on individuals, and leads to a culture of fear of self‐disclosure due to the perception of these programs; this argument is supported by Choflet et al. (2023), whose Google search of ATD programs described programs as punitive, cautioned against self‐report and recommended legal advice before approaching ATD programs. The punitive nature of some current ATD programs can also be linked to societal perceptions of the ‘war on drugs,’ where addiction is a moral failing, and is further compounded by the notion of the impaired nurse being seen as a “liability,” as a participant described in a study of 268 nurses who were Peer Health Assistance Program participants in the United States (Pace et al. 2020). To address these issues, there is an urgent need to research harm reduction as a strategy in ATD programs to improve nurse wellbeing, provide care before individuals are at risk of losing their careers, and to educate and inform the profession on the needs of nurses who use alcohol and/or other drugs.

This position is also reflected in the demands placed on alternative to discipline program participants. For example, many programs require abstinence from ‘mind altering substances’ such as alcohol, even if this was not the focus of the treatment episode. Further, some programs required abstinence from pharmacotherapy and medications for mental health conditions, clearly in contravention of most global treatment guidelines (e.g., Bruneau et al. 2018). With many countries around the world adopting a harm reduction treatment paradigm, where pharmacology is an accepted part of alcohol and other drug treatment, the strict requirement for abstinence as an outcome may not fit. This point is illustrated where opioid use is concerned; as indicated in our literature review, some programs demand abstinence from opioid substitution therapies such as buprenorphine and methadone, which are treatments for opioid dependence that are supported by both evidence and expert consensus (Degenhardt et al. 2023; Kampman and Jarvis 2015; Soyka et al. 2011). Further research is required into continued use of opioid substitution therapies for nurses when entering alternative to discipline programs, especially considering that continuing these medications, rather than demanding cessation as part of an abstinence‐based approach, has been shown to improve treatment outcomes and retention among the general population taking part in 12‐step programs (Klein and Seppala 2019; Monico et al. 2015).

The financial cost of programs was described by participants in this study as a significant burden to participating in an ATD program, especially where limitations are placed on the number of shifts the nurse can work while enrolled in the program. Put simply, an inability to work means no income to pay for ATD programs. Even those with a sponsored component, such as the program described by Ross et al. (2020), required extensive payment for the subsequent 2 years of the program. Although saving a nursing career, ATD programs may leave participants in significant debt or be inaccessible to those with limited financial means.

Although most of the programs included in this review enabled self‐report and voluntary entry, most participants in alternative to discipline or monitoring programs entered after an incident or being reported to their nursing regulator. A study of 280 nurse anaesthetists in the United States (Foli et al. 2022) found that participants were reluctant to self‐report their issues with alcohol and/or other drugs due to a perception that the inevitable conclusion was loss of certification, loss of employment and loss of income. As one participant in this study describes, the fear of law enforcement meant they were going to continue using substances “…until I died of overdose …” The perception of the punitive nature of these programs, combined with a reluctance to report leads to a difficult situation for nurses who wish to seek help for their alcohol and/or drug use.

Of note is the lack of literature describing programs for nurses with an alcohol use disorder, with available published literature in this review describing programs for substance use disorder. While opioid use disorder may be the chief reason for seeking an alternative to discipline program in the United States (Mumba et al. 2019) it may not be in other parts of the world. For example, Australian nurses may seek assistance with alcohol consumption (Searby et al. 2024), meaning a tendency to the use of toxicology to ensure abstinence may not be valid.

### 6.1 Limitations

A substantial limitation of this review is the diverse nature and quality of the literature included. Although some papers evaluated the efficacy of alternatives to discipline programs, most described these programs in terms of their components, and often not completely. In addition, there is no clear definition of these programs, with some papers located during the literature search for this review describing peer support programs with no clear alternative to discipline or monitoring program component. Further, there is a need to align components of alternatives to discipline programs to established evidence for treating alcohol and/or drug use, as the abstinence‐based, twelve‐step dominant model of treatment may not be the optimum approach for nurses beyond the United States.

### 6.2 Relevance for Clinical Practice

Impaired practice among nurses has significant consequences on both nurse wellness and patient safety (Kunyk 2015). ATD programs provide an opportunity for early, non‐disciplinary, confidential, evidence‐based treatment to support recovery from alcohol and/or drug use. Participation in ATD programs not only protects the public, but also the nurse's licence or registration remains unencumbered upon successful completion (Pace et al. 2020; Strobbe and Crowley 2017).

## 7 Conclusion

Nurses with SUD challenges in the United States have had access to ATD programs for decades. These programs have provided an evidence‐based pathway to preserve the nurse's registration and their ability to maintain employment upon completion. This paper identifies common components of ATD programs and explores their applicability to other geographic contexts. Despite the benefits of ATD, little is known about their potential utility outside the United States. As program applicability and efficacy are considered, factors such as program components, flexibility, and cost need to be evaluated further.

## Author Contributions

Each author certifies that their contribution to this work meets the standards of the International Committee of Medical Journal Editors.

## Conflicts of Interest

The authors declare no conflicts of interest.

## Data Availability

Data sharing is not applicable to this article as no new data were created or analyzed in this study.
